# Mindfulness for mediating the relationship between self-control and alexithymia among Chinese medical students: A structural equation modeling analysis

**DOI:** 10.3389/fpsyg.2022.966505

**Published:** 2022-12-13

**Authors:** Chong Liu, Can Cui, Kristin K. Sznajder, Jiana Wang, Xiaoxuan Zuo, Xiaoshi Yang

**Affiliations:** ^1^Department of General Surgery, Shengjing Hospital of China Medical University, Shenyang, Liaoning, China; ^2^Department of Social Medicine, China Medical University School of Public Health, Shenyang, Liaoning, China; ^3^Department of Public Health Sciences, Pennsylvania State University College of Medicine, Hershey, PA, United States

**Keywords:** public health, mental health, medical education, mindfulness, alexithymia

## Abstract

**Backgrounds:**

Medical students are prone to experience alexithymia due to academic work overload, which could increase the prevalence of mental illness such as anxiety and depression. The purpose of our study was to estimate the levels of alexithymia and to explore the relationships between alexithymia, self-control, and mindfulness among medical students.

**Materials and methods:**

From March 18th, 2021 to April 9th, 2021, a cross-sectional study with stratified sampling was carried out in China Medical University, Liaoning Province, China. A total of 1,013 medical students participated in this study. The questionnaires pertaining to the Toronto Alexithymia Scale (TAS-26), the Five Facet Mindfulness Questionnaire (FFMQ), and the Self-control Scale (SCS) were used to assess the levels of alexithymia, mindfulness and self-control. We used Hierarchical Multiple Regression (HMR) and structural equation modeling to explore the mediating role of mindfulness between self-control and alexithymia.

**Results:**

The mean score of alexithymia in medical students was 69.39 ± 9.9. After controlling for confounders, males were more likely to experience alexithymia. Self-control, acting with awareness, describing, and observing in mindfulness were negatively associated with alexithymia (*P* < 0.01). Mindfulness mediated the relationship between self-control and alexithymia (a*b = −0.06, BCa 95% CI: −0.09 to −0.031, Percentile 95% CI: −0.089 to −0.031).

**Conclusion:**

Chinese medical students experienced high levels of alexithymia. Self-control could directly attenuate alexithymia for medical students and indirectly affect alexithymia through the mediating path of mindfulness. Initiatives for self-control ability enhancement should be provided to medical students to combat alexithymia. And interventions on mindfulness training should be developed to prevent from alexithymia and promote their mental health.

## Introduction

The ability to express emotions is an important skill in interpersonal communication for the individuals. Lack of emotional expression may lead to negative mental health outcomes, such as alexithymia. Alexithymia, also known as “emotional deafness”, is a maladaptive psychological phenomenon characterized by a lack of imagination and attention to the inner world, with an inability to identify, verbalize and express one’s own emotions and perceive the feelings of others (Nancy, 2014). Furthermore, alexithymia is considered as a personality trait, rather than a mental illness (Norman et al., 2019). Previous studies reported that alexithymia not only existed in people with mental disorders, but also in the general population, especially among medical students (Lală et al., 2010; Popa-Velea et al., 2017), with the prevalence rate between 16.5 and 49.0% (Alzahrani et al., 2020; Zhang et al., 2020). The high prevalence of alexithymia among medical students has sparked widespread concern about this problem in recent years.

However, medical students are prone to confront with alexithymia during their process of professional studies, bearing the academic pressure to master of a large amount of knowledge, perform well on frequent examinations, and practice clinical cases, as well as intense competition between students (Shapiro, 2011). Medical students with alexithymia always experience difficulties and embarrassment of expression emotions and communicating skills. These challenges not only result in detrimental impacts to their psychological well-being, but also deteriorate their work efficacy, such as information processing, adherence to treatment regimens, medical decision making, and patients satisfaction, which finally affects the quality of care and doctor-patient relationships as well as patients’ health outcomes (Isen et al., 1991; Mersin et al., 2020). The stress-alexithymia hypothesis states that individuals who suffer from alexithymia have difficulties in identifying and dealing effectively with stress in the face of challenging life events (Martin and Pihl, 1985), which is a risk factor for mental health conditions, such as anxiety, depression, burnout, and post-traumatic stress disorder (Zahradnik et al., 2009; Bassols et al., 2014). The affective temperamental dysregulation like alexithymia can possibly contributes to negative emotions such as hopelessness and is an important predictor of suicidality (Serafini et al., 2012). Therefore, in order to decrease the detrimental consequences of alexithymia, it is imperative to examine the prevalence of alexithymia among medical students.

According to self-determination theory, people have a positive tendency for personal growth and adjustment, which is conducive to promoting the integration of one’s individual personality and their mental health (Flannery, 2017). Self-control refers to the capability to consciously overcome impulses, habits, or automatic reactions in order to achieve long-term goals by regulating mental processes and changing inner experiences or situational circumstances (Deborah and Van, 1995). Previous studies have reported that lower levels of self-control were associated with negative behavior problems such as procrastination, aggressive behavior, addictive behavior, and eating disorders (de Ridder et al., 2012). In addition, individuals with lower levels of self-control were more likely to experience difficulties in interpersonal adaptation and increasing risks of anxiety and depression (Tangney et al., 2004). However, to date, the relationship between self-control and alexithymia has not been reported even it has significant implications for mental health.

The alexithymia and major depression are highly associated, however, there are scarcity of non-pharmacological treatments in clinical practice such as enhancing exercise which was shown to be effective both in reducing mortality and treating symptoms of major depression (Martino et al., 2019). Whereas, the positive resources may prevent the risk of mental illness and increase mental well-being for students by enhancing their psychological resources. According to the three axioms, mindfulness is considered as an intention, attitude or attention, emphasizing reduction of real-time control of individual’s emotion, which can contribute to the positive psychological outcomes (Day et al., 2020). Mindfulness is an awareness of the current moment with openness and acknowledgment of each feeling, thought, and emotion in the present time and experiencing the feelings fully without judgment (Cardaciotto et al., 2008). Previous studies have found that mindfulness has a positive association with self-control and negative association with psychological problems such as anxiety, depression, and alexithymia (Hofmann et al., 2010; Williams et al., 2017). Mindfulness is multidimensional and includes acting with awareness, describing, non-judging, non-reactivity, and observing (Carpenter et al., 2019). Acting with awareness involves undivided attention to the current experience. Describing refers to putting one’s inner experiences and feelings into words. Non-judging refers to evaluating one’s thinking and feelings without judgment. Non-reactivity refers to letting one’s inner feelings drift without reacting to them. The definition of observation is the tendency to observe internal and external experiences and is conceptualized as the process or tendency to focus on one’s cognition, emotion, and sensations (Carpenter et al., 2019). De Bruin reported that numerous aspects of mindfulness were associated with a decreased risk of alexithymia (de Bruin et al., 2012). People with high levels of mindfulness have the propensity to identify, understand, and articulate both of their own and others’ current feelings, emotions, and experiences with acceptance, openness, and non-judgment, which contributes to lower levels of alexithymia (Helen and Jennifer, 2017). Further, research has found that mindfulness mediates the relationship between stress and internet addiction among Korea’s general population and mediates the relationship between mindfulness practice and empathy among 264 England students (De la Fuente-Anuncibay et al., 2019; Woo and Jae, 2019). Therefore, we hypothesize that mindfulness mediates the relationship between self-control and alexithymia among medical students.

At the same time, there is a paucity of research exploring the relationships between self-control, mindfulness, and alexithymia among Chinese medical students. In Chinese college students, some studies have shown that there is a significant positive correlation between mindfulness level and self-control (Chen, 2015). Previous studies found that self-control and mindfulness were negatively associated with alexithymia (Fang et al., 2019; Huang et al., 2021), and a close link existed between mindfulness and self-control. Mindfulness training includes the participation of self-awareness and insight perception (Grabovac et al., 2011). The exercise and response of the above two processes require self-control participation. This is mainly due to the attention control and emotional control in the mindfulness training, and the positive emotions generated in the practices themselves all contribute to improve the levels of self-control. Liu (2012) found that the individuals involved in mindfulness meditation had high levels of self-control and mindfulness, and that the mindfulness could predict self-control. In the study of alexithymia, it was shown that positive emotions and an increase in self-awareness could reduce alexithymia, and mindfulness training also increased the positive emotional experience, and avoided negative affection and perceptions, so we hypothesized that mindfulness could alleviate alexithymia (Fan, 2018; Norman et al., 2019). It is of great significance to improve medical students’ mental health and overall well-being in order to address quality of health care delivery and health outcomes for patients. This study sought to address the following hypotheses: (1) Lower sense of self-control is associated with higher alexithymia; (2) Mindfulness has a direct positive impact on lower alexithymia; (3) Mindfulness mediates the relationship between self-control and alexithymia.

## Materials and methods

### Study design

The cross-sectional survey with stratified sampling was performed from March 18th, 2021 to April 9th 2021 in China Medical University and Dalian Medical University in Liaoning province. A self-administered smartphone questionnaire was distributed *via* Wenjuanxing, one of the most popular and professional online platforms in China. In each medical school, eight classes in each grade were randomly selected, with the whole members of the class as the recruitment target. The inclusion criteria were as follows: First year to third year students; could independently complete the online Chinese questionnaire; were able to provide signed informed consent and took part in the study voluntarily. The anonymous questionnaire took about 15 to 20 min to finish. A total of 1,396 medical students completed the questionnaire, resulting in the valid response rate of 88.7%.

### Ethics statement

The Ethics Committee at China Medical University approved this study in accordance with the Helsinki declaration (1989). All participants volunteered to participate in the study and gave informed consent prior to data collection. The survey was conducted anonymously to ensure adequate protection of the respondents’ personal privacy. They were also informed of their right to withdraw from the investigation at any time for any reason.

## Instruments

### Demographic characteristics of participants

Demographic information in this study were collected by the following questions including age (≦19 years or >19 years), gender (male or female), grade (freshman, sophomore, or above), major (clinical medicine or others), monthly income (<3,000 RMB, 3,000–6,000 RMB or > 6,000 RMB), history of disease (yes or no).

### Toronto alexithymia scale

The Toronto Alexithymia Scale (TAS-26) was used to measure the levels of alexithymia. The TAS-26 is a 26 item scale with possible responses using a five-point Likert scale from 1 (strongly disagree) to 5 (strongly agree) (Taylor et al., 1985). Higher scores indicate higher levels of alexithymia. The Cronbach’s alpha of the TAS-26 was 0.759.

### Five facet mindfulness questionnaire

The Five Facet Mindfulness Questionnaire (FFMQ) was employed to evaluate mindfulness among medical students. FFMQ scales had a total of 39 items, consisting of five subscales including acting with awareness, describing, non-judging, non-reactivity, and observing (Baer et al., 2006). The scales were rated on a 5-point Likert-type scale from 1 (Never or very rarely true) to 5 (Very often or always true). Higher scores indicated higher levels of mindfulness. The Cronbach’s alpha of the five facets in this study were 0.912, 0.727, 0.836, 0.776, and 0.867, respectively.

### Self-control scale

The Self-control Scale (SCS) was used to assess the capability of self-control (Tangney et al., 2004). The SCS scale had a total of 13 items which were rated with a 5-point Likert-type scale from 1 (not at all) to 5 (very much). Higher scores imply higher levels of self-control. The Cronbach’s alpha coefficient for the SCS was 0.819, which indicates this scale had a good reliability.

### Statistical analysis

We used SPSS 25.0 and AMOS 17.0 to conduct the statistical analyses. A two-tailed probability value of less than 0.05 was used to test for statistically significant associations. *t*-tests and one way analysis of variances (ANOVAs) were performed to test the differences among categorical variables. A spearman correlation analysis was used to test the relationships between continuous variables including alexithymia, self-control, and mindfulness. Additionally, we used hierarchical multiple regression (HMR) to analyze the incremental contribution of the independent variables and the mediating influence of mindfulness on the relationship between self-control and alexithymia. Alexithymia was included as the dependent variable. Self-control and mindfulness were included as independent variables. All variables were entered by the following three steps: (1) demographic characteristic of medical students; (2) self-control; and (3) mindfulness.

The structural equation model (SEM) was used to test the mediating role of mindfulness in the relationship between self-control and alexithymia. The model fit was tested by the following criteria: χ^2^/df < 5, CFI > 0.90, TLI > 0.90, GFI > 0.90, and RMSEA < 0.08 (James et al., 2006). Bootstrapping with an estimated 5,000 samples was performed to test the mediating role of mindfulness (a × b product) in the relationship between self-control and alexithymia. We calculated bias-corrected and Bias-corrected accelerated 95% confidence interval (Bca 95% CI) and percentile 95% confidence intervals (percentile 95% CI) for a*b estimates. The mediating role was statistically significantly when 95% CI did not include 0 (Wang et al., 2020).

## Results

### Demographic characteristics of the medical students

Demographic characteristics among medical students are summarized in Table 1. Among the 1,238 respondents, the age of the medical students ranged from 18 to 26 years old, with an average age of 19.4 ± 1.1. More than half (66.0%) of students were females, most students (51.5%) were sophomores or above. About two fifths of the students majored in clinical medicine. Nearly two fifths of the medical students’ family (40%) had a monthly income between 3,000 and 6,000 RMB. The majority of students (92.7%) did not report any history of disease.

**TABLE 1:** Demographic characteristics and distributions of alexithymia among medical students (*N* = 1,238).

Variables	*N* (%)	Alexithymia (Mean ± SD)
**Age (years)**		
≤19	705 (56.9)	68.85 ± 9.79
>19	533 (43.1)	70.11 ± 10.11*
**Gender**		
Male	421 (34.0)	71.47 ± 9.32**
Female	817 (66.0)	68.33 ± 10.10
**Grade**		
Freshman	600 (48.5)	69.38 ± 9.70
Sophomore and above	638 (51.5)	69.41 ± 10.19
**Major**		
Clinical medicine	536 (43.3)	68.68 ± 10.39
Other	702 (56.7)	69.95 ± 9.57
**Monthly family income (– Y)**		
<3,000	249 (20.1)	70.86 ± 9.31*
3,000–6,000	495 (40.0)	69.02 ± 9.89
>6,000	494 (39.9)	69.04 ± 10.27
**History of disease**		
No	1148 (92.7)	69.21 ± 9.98
Yes	90 (7.3)	71.83 ± 9.27

*Significant at the 0.05 level (two-tailed). **Significant at the 0.01 level (two-tailed).

Alexithymia scores in the medical students aged over 19 years old were significantly higher than those aged 19 years and below (*P* < 0.05). Male medical students had significantly higher scores for alexithymia than female medical students (*P* < 0.01). Students whose family’s monthly income was under 3,000 RMB were reported to have a higher level of alexithymia (*P* < 0.05).

### Correlations among alexithymia, mindfulness, and self-control

Spearman correlation analyses were conducted to analyze correlations between self-control, mindfulness, and alexithymia, and are presented in Table 2. The results revealed that alexithymia was significantly linked with self-control and the four dimensions of mindfulness (*P* < 0.01). Specifically, acting with awareness, describing, non-judging, and observing were negatively related to alexithymia (*P* < 0.01). Self-control was also found to be negatively related to alexithymia (*P* < 0.01).

**TABLE 2:** The correlations of continuous variables.

	Mean ± SD	1	2	3	4	5	6	7
Alexithymia	69.40 ± 9.95	1						
Self-control	40.56 ± 7.12	−0.415**	1					
Acting with awareness	25.24 ± 5.82	−0.438**	0.604**	1				
Describing	25.50 ± 4.13	−0.520**	0.394**	0.262**	1			
Non-judging	22.64 ± 4.76	−0.168**	0.360**	0.686**	–0.05	1		
Non-reactivity	22.07 ± 3.88	0.042	−0.155**	−0.575**	0.211**	−0.714**	1	
Observing	25.77 ± 5.25	−0.075**	−0.101**	−0.447**	0.331**	−0.682**	0.709**	1

**Significant at the 0.01 level (two-tailed).

### Associated factors of alexithymia

**FIGURE 1:**
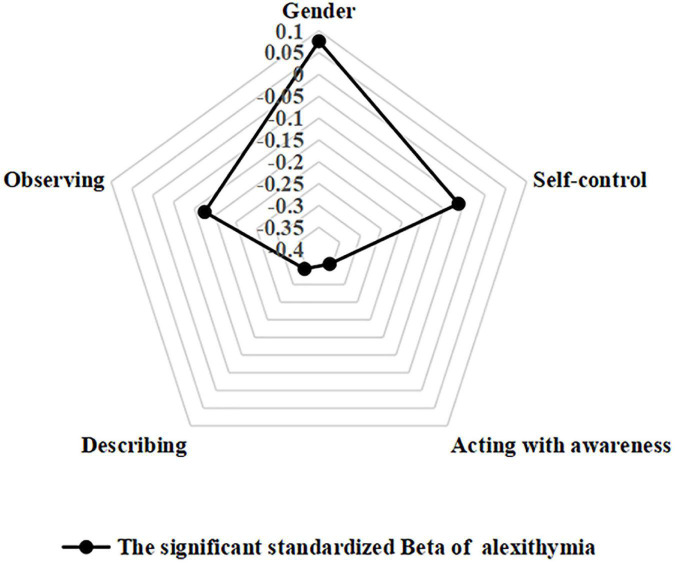
Radar chart of alexithymia among medical students.

**FIGURE 2:**
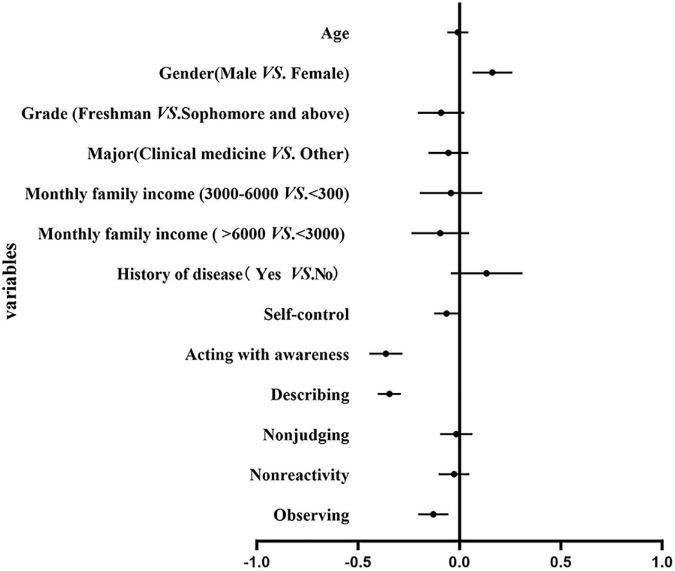
Forest plot of associate factors of alexithymia.

The final results of the hierarchical multiple regression models of alexithymia are presented in Table 3. The final regression model accounted for 40.1% of the total variance, with self-control and mindfulness accounting for 15 and 20% of the total variance. Gender, self-control, and mindfulness were significantly associated with alexithymia. Male medical students had a higher risks of alexithymia (β = 0.162, 95% CI 0.064 to 0.261, *P* < 0.001), while self-control (β = −0.064, 95% CI −0.125 to −0.030, *P* < 0.05) and dimensions of mindfulness including acting with awareness (β = −0.363, 95% CI −0.445 to −0.282, *P* < 0.001), describing (β = −0.346, 95% CI −0.403 to −0.289, *P* < 0.001) and observing (β = −0.129, 95% CI −0.204 to −0.054, *P* < 0.001) decreased the risks of suffering from alexithymia (Figures 1, 2).

**TABLE 3:** The hierarchical linear regression analysis of alexithymia.

Variables	Model 1			Model 2			Model 3		
	β	Standardized β	95% CI	β	Standardized β	95% CI	β	Standardized β	95% CI
**Block 1 Demographic characteristics**									
Age (≤19 vs. >19)	–0.006	–0.006	−0.070, 0.059	–0.007	–0.007	−0.066, 0.052	–0.009	–0.009	−0.060, 0.043
Gender (male vs. female)	0.332**	0.156**	0.210, 0.454	0.274	0.129**	0.162, 0.052	0.162**	0.076**	0.064, 0.261
Grade (Freshman vs. Sophomore and above)	–0.046	–0.023	−0.188, 0.097	–0.079	–0.039	−0.209, −0.034	–0.091	–0.045	−0.206, 0.024
Major (Clinical medicine vs. Other)	−0.135*	−0.067*	−0.258, −0.012	–0.147	−0.073*	−0.260, −0.034	–0.055	–0.027	−0.153, 0.044
Monthly family income (3,000–6,000 vs. <3,000 RMB)	–0.041	–0.020	−0.234, 0.067	–0.026	–0.013	−0.203, 0.151	–0.042	–0.021	−0.196, 0.112
Monthly family income (>6,000 vs. <3,000 RMB)	–0.111	–0.055	−0.289, 0.067	–0.096	–0.047	−0.259, 0.068	–0.095	–0.047	−0.237, 0.047
History of disease (Yes vs. No)	0.252*	0.065*	0.032, 0.472	0.146	0.038	−0.056, 0.349	0.134	0.034	−0.043, 0.310
**Block 2 Self-control**				–0.394	−0.393**	−0.447, −0.341	−0.064*	−0.064*	−0.125, −0.003
**Block 3 mindfulness**									
Acting with awareness							−0.363**	−0.358**	−0.445, −0.282
Describing							−0.346**	−0.344**	−0.403, −0.289
Non-judging							–0.016	–0.015	−0.095, 0.064
Non-reactivity							–0.027	–0.027	−0.103, 0.048
Observing							−0.129**	−0.125**	−0.204, −0.054
**R^**2**^**		0.050			0.200			0.200	
**Adjusted R^**2**^**		0.043			0.193			0.393	
**△R^**2**^**		0.050			0.150			0.201	

*Significant at the 0.05 level (two-tailed). **Significant at the 0.01 level (two-tailed).

### Mindfulness as a mediator between self-control and alexithymia

**FIGURE 3:**
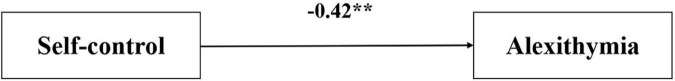
Standardized solutions for the structural equation model of self-control and alexithymia.

Table 4 and Figure 3 depict the pathway coefficients of the SEM model, indicating the direct effects of self-control on alexithymia. As hypothesized, self-control affected alexithymia positively (c = −0.42, *P* < 0.001) with good model fit indicators (χ^2^/df = 4.078 < 5, GFI = 0.983, AGFI = 0.954, CFI = 0.983, TLI = 0.958, RMSEA = 0.05). On the basis of good model fitting, the Bootstrap program was used to repeat the sample for 5,000 times. The results show that the path coefficients between self-control, mindfulness and alexithymia are significant, and self-controls are positively with mindfulness (β = 0.39, *P* < 0.001). Mindfulness are negatively with alexithymia (β = −0.15, *P* < 0.001). Self-control were significantly associated with alexithymia (β = −0.36, *P* < 0.001).

**TABLE 4:** The path coefficients of the mediation model.

			B	β	S.E.	C.R.	*P*
Mindfulness	←	Self-control	0.49	0.39	0.018	26.746	<0.001
Alexithymia	←	Mindfulness	–0.17	–0.15	0.035	–4.804	<0.001
Alexithymia	←	Self-control	–0.50	–0.36	0.041	–12.292	<0.001

B, the unstandardized path coefficient; β, the standardized path coefficient; S.E., the standard error; C.R., the critical ratio; P, the significance level.

**FIGURE 4:**
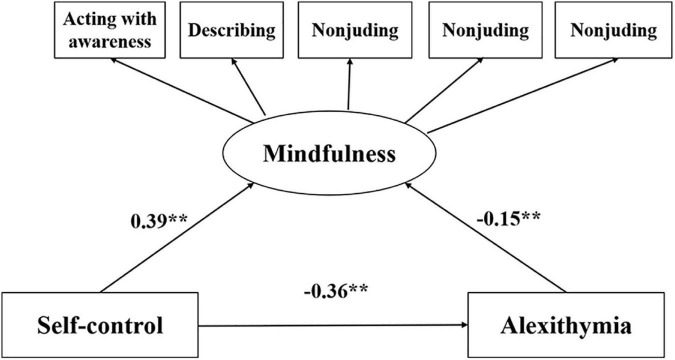
Structural equation modeling of the mediating role of mindfulness in the relationship between self-control and alexithymia.

Figure 4 depicts the mediating role of mindfulness in the relationship between self-control and alexithymia. When mindfulness is included as a mediator in the relationship between self-control and alexithymia, the path coefficient from self-control to alexithymia significantly decreases (c = −0.36, *P* < 0.001) (a*b = −0.06, BCa 95% CI: −0.09, −0.031, Percentile 95% CI:−0.089 to −0.031) with good model fit indicators (χ^2^/df = 1.513 < 5, GFI = 0.97, AGFI = 0.990, CFI = 0.977, TLI = 0.998, RMSEA = 0.02). The results of the SEM indicate that mindfulness has a significant mediating effect in the relationship between self-control and alexithymia.

## Discussion

Our study indicates that Chinese medical students experience high levels of alexithymia. Self-control and mindfulness were negatively related with alexithymia, which could promote to alleviate alexithymia. And mindfulness mediated the relationship between self-control and alexithymia. Self-control could directly attenuate alexithymia for medical students and indirectly through the mediating path of mindfulness to alexithymia. The mean score of alexithymia among medical students in this study was 69.39 ± 9.99, which was similar with Chinese Xinxiang medical students (65.70 ± 7.98) (Ren, 2006), however, the alexithymia in our study was higher than the levels of Turkish nursing students (50.09 ± 8.22) (Mersin et al., 2020). The results in our study indicate that male students reported a higher level of alexithymia than female students. The males were less likely to express their vulnerable feelings and emotions and were prone to have emotional containment, which may increase the risk of suffering from alexithymia (Stanton et al., 2000). Furthermore, our study indicates that self-control could ameliorate the risks of alexithymia, and mindfulness, serving as a partial mediator, could enhance the negative influence of low self-control on alexithymia.

To the best of our knowledge, this study is the first to test the mediating role of mindfulness in the relationship between self-control and alexithymia. This study goes beyond previous studies by revealing that the medical students with poorer self-control report higher levels of alexithymia. People with high self-control have a more healthy pace of life (such as healthy diet and regular exercise), which has been shown to be effective both in reducing mortality and treating symptoms of major depression. An extensive literature research found the detrimental effects of low levels of self-control on individuals’ overall well-being, such as deterioration of personal relationships and triggering a high risk of alexithymia (Tangney et al., 2004). In addition, the empirical relationship between self-control and alexithymia has been confirmed by neurological studies, which found sufficient self-control could relieve one’s stress, control impulsive behavior and minimize aggregated negative emotions by enhancing the functioning of the parasympathetic nervous system, which reduced heart rate and heart rate variability (Hofmann et al., 2014).

Our survey found that mindfulness has a negative influence on alexithymia, which was consistent with previous research (de Bruin et al., 2012). Mindfulness could improve individuals’ ability to identify emotions and express them verbally through enhancing their internal awareness and emphasizing living in the present-moment with non-judgmental and non-interfering attitudes (Lutz et al., 2008). A higher level of mindfulness can increase the cortical thickness of the brain when individuals suffer from unbearable emotions, which could increase the ability to process emotional change and mental well-being (Lutz et al., 2008). A large number of studies have demonstrated that higher levels of mindfulness are associated with lower levels of alexithymia and higher levels of mental well-being (Baer et al., 2006; Morgan and Koa, 2010). Therefore, it is imperative for medical students to improve their mindfulness to reduce the levels of alexithymia, which could be done through mindfulness practice and training.

Previous studies on alexithymia mainly focuses on clinical medicine and the adults or the chronic patients, and rarely explore the relationship between self-control and alexithymia (Dong et al., 2021). The evaluation of alexithymia among medical students needs to be further improved. With regard to hypotheses (3), mindfulness mediates the relationship between self-control and alexithymia. Our study found that mindfulness partially mediated the relationship between self-control and alexithymia. Self-control not only directly influenced alexithymia among medical students, but also indirectly influenced alexithymia by enhancing mindfulness, especially in the dimensions including acting with awareness, describing, and observing. In our study, the above three dimensions of mindfulness were negatively associated with alexithymia. Specifically, acting with awareness, which focused on the present time rather than rumination on the past, alleviated the risk of alexithymia. Similar findings reported by Joseph et al. indicated that acting with awareness was the dimension that was associated with lower levels of affective symptoms and alleviation the risk of alexithymia (Carpenter et al., 2019). Furthermore, this study also found that describing was negatively associated with alexithymia. A higher level of describing might enhance individuals’ ability to express their inner emotions in a positive and accurate way, which could help individuals mitigate the risks of alexithymia and promote mental well-being in stressful environments (Helen and Jennifer, 2017). These results were in concordance with previous research from Hong Kong, which demonstrated that describing was significantly negatively related to mental well-being (Hou et al., 2014). In addition, observing was found to have a negative effect on alexithymia in our study. Participants with a higher sense of observation were more likely to pay attention to internal and external stimuli rather than focus rigidly on specific negative stimuli, such as unpleasant events or thoughts (Tamanaeifar et al., 2021), which decreases the likelihood of alexithymia. Medical students may experience low levels of mindfulness and an increasing risk for alexithymia due to lack of self-control, which ultimately contributes to a higher risk of alexithymia.

The results of this study suggest that the medical education of the students should focus not only on the cultivation of professional knowledge, but also on cultivating their self-control ability. Also, mindfulness intervention should be provided to cope with difficulties and stressful environments. These results will add evidence to the existing literature and serve as baseline data for policy makers, mental health professionals, and clinicians to assist in the development and implementation of evidence-based interventions and initiatives for alexithymia and mental health among Chinese medical students.

## Limitations

There are several limitations in the present study. Firstly, due to the cross-sectional design based on self-reported measurements, it is difficult to draw conclusions about the causal relationships between self-control, mindfulness, and alexithymia. Longitudinal studies are essential to explore the causal relationships in the future. Secondly, since the survey was conducted in two medical schools in the province of Liaoning, China, the generalizability to other populations was limited. Further studies are needed to examine the applicability of the present results among populations in different settings including diverse cultural and social backgrounds. Thirdly, the results of the study may be limited by the COVID-19 pandemic outbreak and other unmeasured confounders, as the study was conducted between March 2021 and April 2021. Fourthly, selection bias cannot be excluded, as the survey was conducted online and the participants were only smartphone users. Finally, the baseline characteristics did not include factors such as residence, whether to be an only child, and family type, which should be added in the future studies. Future studies should examine other social contextual factors to better understand the impact of psychosocial variables on alexithymia among Chinese medical students. Additional longitudinal research is warranted to confirm these findings and more psychosocial factors should be included for exploration of mechanisms of alexithymia among medical students.

## Conclusion

Chinese medical students experienced high levels of alexithymia. Our study indicates that Chinese medical students experience high levels of alexithymia. Self-control and mindfulness were negatively associated with alexithymia. Mindfulness was observed to enhance the positive influence of self-control on alexithymia through the mediating effect of acting with awareness, describing, and observing. Therefore, promoting self-control and training on mindfulness are necessary to reduce the risks of alexithymia and promote mental well-being among medical students.

## Data availability statement

The raw data supporting the conclusions of this article will be made available by the authors, without undue reservation.

## Ethics statement

The studies involving human participants were reviewed and approved by China Medical University. The patients/participants provided their written informed consent to participate in this study.

## Author contributions

CL contributed to the acquisition and analysis of the data. CC contributed to the acquisition and analysis of the data, drafting, and revision of the manuscript. KS contributed to revision of the manuscript and provided the English edits. JW contributed to the conception and design of the manuscript. XZ contributed to revision of the manuscript format. XY contributed to revision the final version and are guarantors of the manuscript. All authors read and approved the final manuscript.
